# Riding the COVID Waves: Clinical Trends, Outcomes, and Remaining Pitfalls of the SARS-CoV-2 Pandemic: An Analysis of Two High-Incidence Periods at a Hospital in Northern Italy

**DOI:** 10.3390/jcm10225239

**Published:** 2021-11-11

**Authors:** Marina Sartini, Filippo Del Puente, Martino Oliva, Alessio Carbone, Elisabetta Blasi Vacca, Andrea Parisini, Silvia Boni, Nicoletta Bobbio, Marcello Feasi, Alessandra Battistella, Emanuele Pontali, Maria Luisa Cristina

**Affiliations:** 1 Department of Health Sciences, University of Genova, Via Pastore 1, 16132 Genova, Italy; cristinaml@unige.it; 2 Operating Unit (S.S.D. U.O.) Hospital Hygiene, Galliera Hospital, Mura delle Cappuccine 14, 16128 Genoa, Italy; martino.oliva@galliera.it (M.O.); alessio.carbone@galliera.it (A.C.); 3 Department of Infectious Diseases, Galliera Hospital, Mura delle Cappuccine 14, 16128 Genoa, Italy; elisabetta.blasi@galliera.it (E.B.V.); andrea.parisini@galliera.it (A.P.); silvia.boni@galliera.it (S.B.); nicoletta.bobbio@galliera.it (N.B.); marcello.feasi@galliera.it (M.F.); emanuele.pontali@galliera.it (E.P.); 4 Medical Service Management, Galliera Hospital, Mura delle Cappuccine 14, 16128 Genoa, Italy; alessandra.battistella@galliera.it

**Keywords:** SARS-CoV-2, outcome, clinical trends

## Abstract

Background. Italy was the first western country to face an uncontrolled outbreak of SARS-CoV-2 infection. The epidemic began in March 2020 within a context characterised by a general lack of knowledge about the disease. The first scientific evidence emerged months later, leading to treatment changes. The aim of our study was to evaluate the effects of these changes. Methods. Data from a hospital in Genoa, Italy, were analysed. Patients deceased from SARS-CoV-2 infection were selected. Data were compared by dividing patients into two cohorts: “phase A” (March–May 2020) and “phase B” (October–December 2020). Results. A total of 5142 patients were admitted. There were 274 SARS-CoV-2-related deaths (162 phase A and 112 phase B). No differences were observed in terms of demographics, presentation, or comorbidities. A significant increase was recorded in corticosteroid use. Mortality was 33.36% during phase A, falling to 21.71% during phase B. When subdividing the trend during the two phases by age, we found a difference in people aged 65–74 years. Conclusions. There is scarce evidence regarding treatment for SARS-CoV-2 (especially for severe infection). However, treatment changes improved the prognosis for people under the age of 75. The prognosis for older people remains poor, despite the improvements achieved.

## 1. Introduction

The SARS-CoV-2 infection was first reported in China in 2019 and subsequently spread to the rest of the world. It had a significant negative impact on Italy, both in terms of morbidity and mortality, causing more than 4 million cases (7% of the population) and more than 132,000 deaths as of October 2021 [1].

The magnitude of the impact was primarily due to two factors. Firstly, the Italian population is the oldest in Europe. It has been repeatedly cited in the literature that age is possibly the most crucial aspect associated with SARS-CoV-2 infection severity [2]. Secondly, Italy was the first western country to suffer an uncontrolled outbreak. This meant that Italian healthcare providers and the government had to develop control and treatment measures rapidly, at a time when scientific evidence on the treatment of SARS-CoV-2 infection was still lacking [3].

During that period (until approximately April/May 2020), the lack of good-quality scientific evidence led international bodies to draw up therapeutic guidelines, such as the use of chloroquine and the relative contraindication of corticosteroid use, which were later revised or abandoned completely due to the therapeutic results [4]. All these factors may well have contributed to making the clinical outcomes worse, especially during the first phase of the pandemic [5,6,7].

In addition to these issues, we must also consider the Italian Health Service’s lack of preparation to handle the rapid development of a healthcare crisis, which led to the need to transform care by adapting it to the demands of the growing number of SARS-CoV-2-infected patients [8,9]. Indeed, despite extensive studies on the COVID-19 pandemic and its effect on various pathological conditions [10,11,12,13], there are no comprehensive data available regarding the effect of the pandemic on hospital organisation and outcomes during the most crucial phases of the epidemic.

In Italy, the Istituto Superiore di Sanità (ISS) published a report on 28 April 2021 listing the characteristics of 118,592 deceased and SARS-CoV-2 positive patients through the Integrated Surveillance COVID-19 system [14]. The ISS considered three phases: the first wave (March–May 2020), the low-incidence phase (June–September 2020), and the second wave (October 2020–April 2021), of which the latter is still ongoing at the time of writing [15].

In our work, we evaluated the epidemiological trend during two clinically significant phases (March–May 2020, defined Phase A, and October–December 2020, defined Phase B) of the Italian scenario at a hospital in the metropolitan area of Genoa. In between these two phases, the treatment of SARS-CoV-2 infection underwent several advances based on new scientific evidence, transforming the clinical course for patients, and bringing about improved care [16,17,18].

The objectives of our study were: (1) to evaluate the baseline characteristics, principal comorbidity and number of comorbidities (calculated using the Charlson Comorbidity Index), and management differences for patients who died of SARS-CoV-2 infection during Phases A and B; (2) to highlight any differences in clinical outcomes and mortality between patients hospitalised during Phases A and B; and (3) to understand which patient categories made most use of therapeutic updates and, conversely, which populations did not derive the desired benefit from therapeutic advances, by analysing the conditions of patients who died during the two phases.

## 2. Materials and Methods

The study was conducted in a nationally renowned, highly specialised northern Italian hospital organised by treatment intensity. The hospital is made up of pavilions and has 458 beds (mainly in 3- and 4-bed rooms), with more than 15,000 routine admissions per year, and more than 8600 medical procedures in outpatient and day surgery settings.

During the pandemic, the management of the hospital was adapted to meet the demand for admission of greater numbers of SARS-CoV-2-infected patients by reducing the space available for SARS-CoV-2-negative patients. SARS-CoV-2-infected patients were accommodated in 2- and 3-bed rooms after the triage, which was performed in a buffer zone where patients with characteristics deemed suspicious for SARS-CoV-2 infection were isolated until sufficient data were collected.

All patients discharged between 1 March and 31 May 2020 (Phase A) and between 1 October and 31 December 2020 (Phase B) were enrolled.

All patients who died during the two periods were then selected. We recorded biographical data, medical history, vital parameters and laboratory tests, treatment, and clinical course for this cohort. The outcome of the nasopharyngeal molecular swab (RT-PCR method) performed to diagnose SARS-CoV-2 in hospitalised patients was also recorded. The study was conducted in accordance with the Declaration of Helsinki on ethical principles for medical research. The data were anonymised and subsequently evaluated following our hospital policy for safeguarding patient privacy.

In accordance with international guidelines, SARS-CoV-2 infection was diagnosed either via a RT-PCR test using a nasopharyngeal swab, or in the event of a negative or inconclusive RT-PCR test, by means of clinical and radiological data such as the presence of a concordant clinical picture and a thorax CT scan that could be directly attributed to a viral infection, in the absence of other possible aetiologies of pulmonary infiltration [19,20,21,22,23].

Between Phase A and Phase B, treatment changed and was adapted on the basis of new data available in the literature and guidelines regarding the effectiveness of SARS-CoV-2 treatment. As will be highlighted in Section 3, corticosteroid therapy would be more extensively used. Among the patients analysed, the following were excluded: (1) patients with asymptomatic infection; (2) patients whose death was not directly attributed to SARS-CoV-2 infection.

### Statistical Analysis

All the patient characteristics were presented as mean with standard deviation, median and range for continuous variables, and expressed as absolute values along with percentages for categorical variables. As the data did not display a normal distribution, every possible numerical transformation of the data was evaluated. As none of these was able to reduce the effect of skewness, the data were analysed by means of non-parametric tests. The Wilcoxon test was used to compare means, while the Chi-squared test was used to assess independence between variables. Kaplan–Meier curves and log-rank tests were used to analyse survival times for comparisons between groups. All tests were two-sided, and a p-value of less than 0.05 was considered statistically significant. All statistical analyses were performed using Stata/SE 14.2 software (StataCorp LP, College Station, TX, USA).

## 3. Results

A total of 5142 patients were hospitalised during the two Phases (2001 during Phase A and 3141 during Phase B), their mean age was 67 years (median 73, range 0–101) during Phase A and 63 years (median 68, range 0–100) during Phase B. Female sex prevalence was 48.78% during Phase A and 48.01% during Phase B.

Out of these patients, 274 died due to SARS-CoV-2 (162 during Phase A and 112 during Phase B). In these cases, the diagnosis was achieved via an RT-PCR test using a nasopharyngeal swab in 149/162 patients during Phase A and 108/112 patients during Phase B. No demographic difference was observed between patients who died during the two phases (Table 1).

In greater detail, the mean age was 81 years (median 82, range 58–99) during Phase A, while it was 83 (median 85, range 50–98) during Phase B. Female sex prevalence was 40.12% during Phase A and 45.54% during Phase B.

The comorbidity rate also did not differ significantly between the two phases, with only diabetes mellitus prevalence being significantly higher during Phase A. The Charlson Comorbidity Index score and other comorbidities did not differ significantly. In greater detail, the mean Charlson Comorbidity Index score was 5.44 (median 5, range 1–10) during Phase A and 5.63 (median 5, range 1–13) during Phase B.

During Phase A, diagnosis via nasopharyngeal swab was achieved in 0–4 days (median 0, mean 0.78 days). During Phase B, diagnosis was achieved in 1–9 days (median 1, mean 1.55 days).

The patients’ clinical symptoms and laboratory data were evaluated at presentation. Both clinical symptoms and data did not differ significantly, with the sole exception of procalcitonin (higher during Phase A, *p* = 0.024) and D-dimer (higher during Phase B, *p* = 0.029). In terms of clinical presentation, no other differences were observed between vital parameters at baseline (Table 2).

Laboratory data and vital parameter trends were also evaluated using the last data available per patient. We observed a significant difference in these cases, especially as regards the leukocyte and neutrophil count, which significantly increased during Phase B (*p*-values respectively, 0.0049 and 0.087). Meanwhile, the C-reactive protein, procalcitonin fibrinogen, and lymphocyte count trends did not differ significantly.

This difference could be explained by the extended use of corticosteroids during Phase B. Treatment with cortisone derivatives was used to a greater extent and for a higher number of patients during Phase B (70/162 (43%) during Phase A and 94/112 during Phase B (84%)) (Pearson Chi2 = 45.6914, Pr = 0.000) and for a longer duration. In greater detail, the mean corticosteroid dosage was 558.67 mg of prednisone equivalent (median 346.5, range 10–2920) during Phase A, while it was 981.64 mg of prednisone equivalent (median 760, range 20–4950) during Phase B. The mean treatment duration was 8.39 days (median 5.5, range 1–47) during Phase A, while it was 13.37 (median 9, range 1–105) during Phase B.

We also observed a significant difference in hydroxychloroquine and remdesivir use between the two phases, the first being used exclusively during Phase A and the latter being used exclusively during Phase B, albeit in a minority of patients (40/112).

Unsurprisingly, support treatment and ventilation did not differ significantly between Phases A and B. Only a slight increase in Venturi mask ventilation was observed during Phase B.

The mean hospitalisation period did not differ significantly among people who died due to SARS-CoV-2 infection during the two phases, with a mean of 16.14 days (median 10; range 1–144) during Phase A and 15.90 days (median 12, range 2–77) during Phase B.

**Figure 1:**
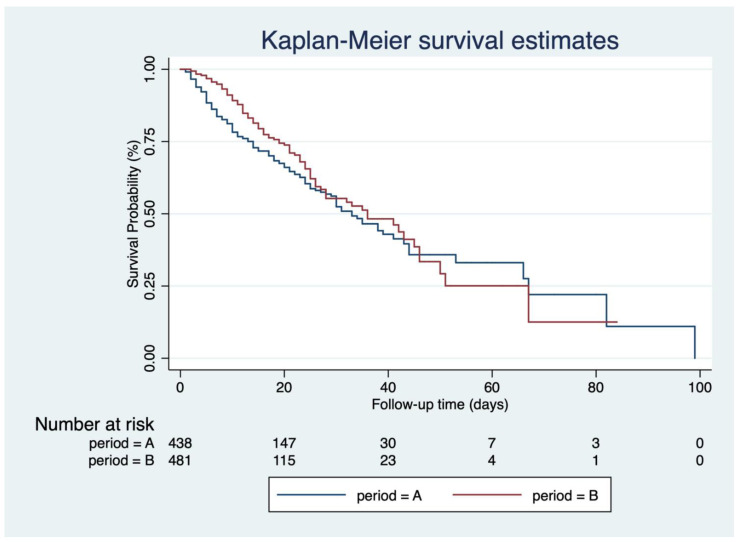
Kaplan–Meier overall survival estimates for SARS-CoV-2-infected patients.

In terms of clinical outcomes, mortality due to both SARS-CoV-2 infection and other causes fell significantly between Phase A and Phase B. Specifically, mortality due to severe SARS-CoV-2 infection during Phase A was 33.36% and 21.71% during Phase B (*p* < 0.001). In comparison, mortality due to non-SARS-CoV-2 infection causes during Phase A was 6.7% and 3.8% during Phase B (*p* < 0.001). Mortality due to SARS-CoV-2 infection was analysed by Kaplan–Meier, which showed a significant difference between the two phases (log-rank test: *p* = 0.022) (Figure 1).

**Figure 2:**
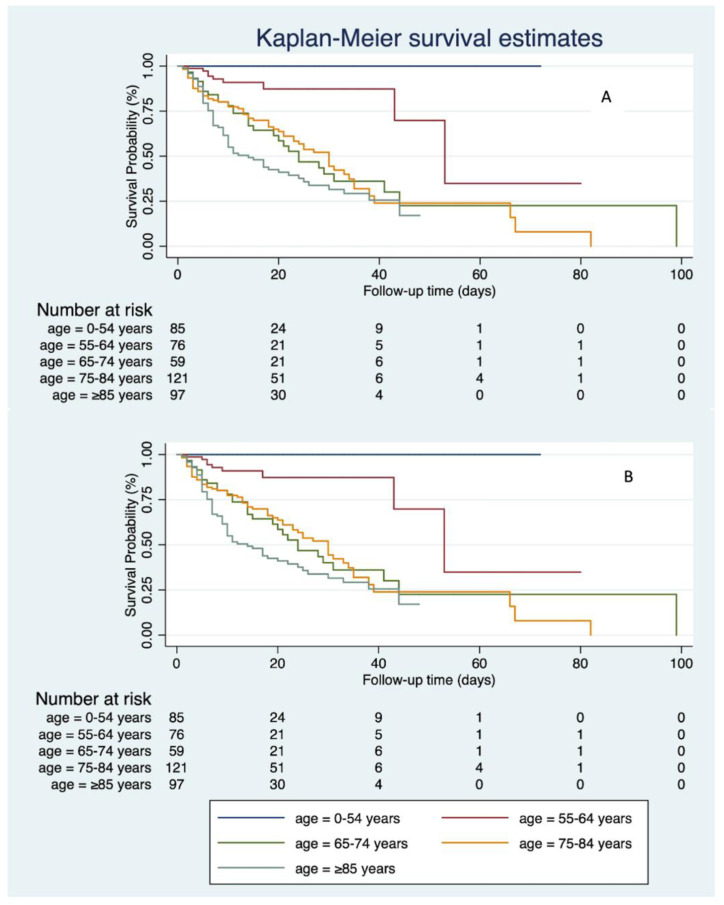
Kaplan–Meier survival estimates for SARS-CoV-2-infected patients divided by age group in the two periods: phase A (**A**) and phase B (**B**).

Due to advanced age being one of the principal risk factors for death in SARS-CoV-2-infected patients, mortality was evaluated in different age groups using the Kaplan–Meier evaluation (Figure 2).

According to this analysis, the clinical outcome improved primarily in patients aged between 65 and 74 years. The difference observed in the mortality curves in this age group was statistically significant (log-rank test: *p* = 0.0264).

Regarding patients < 65 years, the trend continued to produce positive clinical outcomes, while for patients > 75 years, the trend continued to result in mixed clinical outcomes, despite a general improvement observed during Phase B.

## 4. Discussion

As our knowledge of SARS-CoV-2 infection progresses, the clinical outcomes of SARS-CoV-2 infection have improved, especially—in our experience so far—for patients between 65 and 74 years of age. As we highlighted in our paper, we could say that we observed that the same epidemic was repeated, given that the clinical condition at baseline, comorbidities, and laboratory values were found to overlap significantly between the two phases analysed.

Improvements in patient management are apparent in the reduction in mortality, the increased average age at death, and the increase in hospital admissions. This improvement was primarily due to the extensive use of corticosteroid therapy, which almost doubled between the two phases. It is also worth noting that this treatment adjustment had already taken place towards the end of Phase A, improving the clinical outcome for a proportion of patients admitted during that period. These data confirm the results of the several clinical trials that demonstrated the usefulness of corticosteroid treatment in patients with SARS-CoV-2 infection requiring ventilatory support [24]. However, it should be noted that our work does not include data regarding the management of patients prior to hospitalisation and we cannot infer data regarding the population that did not require hospital admission due to medical care received from general practitioners. On the other hand, as clinical presentation did not differ between the two phases, it seems likely that treatments administered by GPs prior to hospitalisation may not have played a primary role in safeguarding the portion of the population that ultimately required hospital care. Lastly, we were unable to gather sufficient evidence regarding pharmacological treatments that were later recommended, such as remdesivir, or supportive therapy, such as the use of NIV [25,26], primarily because they were only used rarely during the first period (as in the case of remdesivir) and because, in case of NIV, its use was only permitted in limited settings (ICU, Infectious Diseases Unit, and sub-critical medical care).

Other informative work published during the epidemic has focused on studying patient trends in individual hospital settings, either on a regional or national level, or focusing solely on ICU patients [16,27,28,29,30,31]. However, to the best of our knowledge, we can say that this study did not limit itself to analysing patient characteristics at baseline or general hospital admission trends, instead it analysed a portion of its patients comprehensively, evaluating their trends and clinical outcome, as well as providing information on therapy and management. Unfortunately, we are unable to draw conclusions regarding the emergence of viral variants as these data were not available to our analysis.

From our perspective, two worrying factors have emerged: firstly, the general lack of preparation observed during the first phase of the pandemic due to the lack of resources and errors embedded in treatment guidelines; and secondly, the lack of clinical improvement in older patients.

Our observations do not currently allow us to provide any answers as to why such a significant part of the population remains subject to a high proportion of unfavourable outcomes. This finding has been the subject of other studies, but for now, there is only an epidemiological correlation [2,32,33,34]. The impact that will be had by new therapeutic systems (monoclonal antibodies; immunomodulatory therapies; antivirals such as remdesivir, which was used in only a proportion of patients during phase B; and vaccinations) or new viral variants, remains to be evaluated and should be the target of new studies and clinical trials designed to gather information regarding specific subpopulations (such as elderly patients or vaccinated and unvaccinated patients).

In view of the limitations inherent to our study (its retrospective nature, the analysis based on deceased patients, and the absence of information on treatment prior to admission), we are able to conclude that, although we are far away from having a so-called “silver bullet” for the SARS-CoV-2 infection, the progressive improvement in our knowledge has allowed for a sharp increase in hospitalisations and an improvement in the overall outcome of patients hospitalised (for whatever reason), despite the comorbidities and advanced age of the patients referred to our hospital.

## Figures and Tables

**Table 1:** Demographic data and comorbidities of patients who died due to SARS-CoV-2 infection.

	Phase A	Phase B	*p*
Number of patients	162	112	
**Age (years)**			0.1121
Mean ± S.D.	81 ± 9	83 ± 9	
Median	82	85	
Range	58–99	50–98	
**Gender (*n* (%))**			0.373
Female	65 (40.12)	51 (45.54)	
Male	97 (59.88)	61 (54.46)	
**CCI**			0.6867
Mean	5.44	5.63	
Median	5	5	
Range	1–10	1–13	
**Diabetes (*n* (%))**			0.024
Yes	59 (36.42%)	26 (23.21%)	
No	103 (63.58%)	86 (76.79%)	
**Cardiopathy (*n* (%))**			0.478
Yes	75 (46.30%)	47 (41.96%)	
No	87 (53.70%)	65 (58.04%)	
**COPD (*n* (%))**	31 (19.14%)		0.789
Yes	131 (80.86%)	20 (17.26%)	
No		92 (82.74%)	
**Liver disease (*n* (%))**			0.594
Yes	4 (2.47%)	4 (3.57%)	
No	158 (97.53%)	108 (96.43%)	
**Nephropathy (*n* (%))**			0.38
Yes	40 (24.79%)	33 (29.46%)	
No	122 (75.21%)	79 (70.54%)	
**Oncology (*n* (%))**			0.463
Yes	17 (10.49%)	15 (13.49%)	
No	145 (89.51%)	97 (86.51%)	

**Table 2:** Vital Parameters.

Admission Data						
	Phase A			Phase B		
	Mean ± SD	Median	Range	Mean ± SD	Median	Range
**pO_2_** (mmHg)	69 ± 30	63.75	24–211	66 ± 31	55	24–226
**SatO_2_** (%)	88 ± 12	93	32–100	89 ± 10	92	35–100
**PCR** (mg/dL)	10.51 ± 9.34	7.85	0.05–38.7	9.49 ± 8.33	7.0	0.07–35.26
**PCT** (ng/mL) *	3.59 ± 13.28	0.38	0.03–100	2.86 ± 14.14	0.26	0.02–100
**Fibrinogen** (mg/dL)	549 ± 180	513	233–1188	516 ± 206	492	231–1100
**D–dimer** (ng/mL) *	6219 ± 14,765	2350	336–131,466	6544 ± 16,341	1716	305–128,926
**Leukocytes** (10^9^/L)	11.20 ± 6.91	9.46	0.15–44.54	10.25 ± 5.72	9.01	0.07–169.73
**Neutrophils** (10^9^/L)	9.27 ± 6.59	7.67	0.03–42.24	8.51 ± 5.26	7.20	0.79–32.71
**Lymphocyte** (10^9^/L)	1.19 ± 1.38	0.91	0.1–12.48	1.03 ± 0.84	0.84	0.22–6.95
**Last Evaluation Available**						
	**Phase A**			**Phase B**		
	**Mean ± SD**	**Median**	**Range**	**Mean ± SD**	**Median**	**Range**
**pO_2_** (mmHg)	70 ± 32	62	28–217	78 ± 61	62	21–475
**SatO_2_** (%)	87 ± 13	91	43–100	90 ± 8	92	60–100
**PCR** (mg/dL)	12.80 ± 9.41	10.82	0.42–54.65	10.48 ± 9.05	7.91	0.53–38.0
**PCT** (ng/mL)	5.52 ± 15.92	0.58	0.12–100	3.36 ± 4.98	1.25	0.53–20.2
**Fibrinogen** (mg/dL)	528 ± 224	429	180–1208	538 ± 236	521	0–20
**D–dimer** (ng/mL)	6732 ± 10,511	2573	169–55,824	7592± 13,550	2321	591–67,876
**Leukocytes** (10^9^/L)	12.10 ± 6.36	10.21	0.16–33.17	15.58 ± 10.35	14.19	3.97–82.77
**Neutrophils** (10^9^/L)	10.57 ± 6.21	8.88	0.1–30.93	14.04 ± 9.45	12.89	3.51–76.4
**Lymphocyte** (10^9^/L)	1.03 ± 2.05	0.76	0–20.32	0.87 ± 1.15	0.61	0.08–94

* *p* < 0.05.

## Data Availability

The data presented in this study are available on motivated request from the corresponding author.

## Abbreviations

RT-PCR	Reverse transcription of polymerase chain reaction
CCI	Charlson Comorbidity Index
COPD	Chronic obstructive pulmonary disease
pO_2_	Partial pressure of arterial oxygen
SatO_2_	Peripheral oxygen saturation
PCR	C-reactive protein
PCT	Procalcitonin
ACE	Angiotensin-converting enzyme
